# Differential Acute Postprandial Effects of Processed Meat and Isocaloric Vegan Meals on the Gastrointestinal Hormone Response in Subjects Suffering from Type 2 Diabetes and Healthy Controls: A Randomized Crossover Study

**DOI:** 10.1371/journal.pone.0107561

**Published:** 2014-09-15

**Authors:** Lenka Belinova, Hana Kahleova, Hana Malinska, Ondrej Topolcan, Jindra Vrzalova, Olena Oliyarnyk, Ludmila Kazdova, Martin Hill, Terezie Pelikanova

**Affiliations:** 1 Institute for Clinical and Experimental Medicine, Prague, Czech Republic; 2 First Faculty of Medicine, Charles University, Prague, Czech Republic; 3 University Hospital in Pilsen, Pilsen, Czech Republic; 4 Institute of Endocrinology, Prague, Czech Republic; Weill Cornell Medical College Qatar, Qatar

## Abstract

**Background:**

The intake of meat, particularly processed meat, is a dietary risk factor for diabetes. Meat intake impairs insulin sensitivity and leads to increased oxidative stress. However, its effect on postprandial gastrointestinal hormone (GIH) secretion is unclear. We aimed to investigate the acute effects of two standardized isocaloric meals: a processed hamburger meat meal rich in protein and saturated fat (M-meal) and a vegan meal rich in carbohydrates (V-meal). We hypothesized that the meat meal would lead to abnormal postprandial increases in plasma lipids and oxidative stress markers and impaired GIH responses.

**Methods:**

In a randomized crossover study, 50 patients suffering from type 2 diabetes (T2D) and 50 healthy subjects underwent two 3-h meal tolerance tests. For statistical analyses, repeated-measures ANOVA was performed.

**Results:**

The M-meal resulted in a higher postprandial increase in lipids in both groups (p<0.001) and persistent postprandial hyperinsulinemia in patients with diabetes (p<0.001). The plasma glucose levels were significantly higher after the V-meal only at the peak level. The plasma concentrations of glucose-dependent insulinotropic peptide (GIP), peptide tyrosine-tyrosine (PYY) and pancreatic polypeptide (PP) were higher (p<0.05, p<0.001, p<0.001, respectively) and the ghrelin concentration was lower (p<0.001) after the M-meal in healthy subjects. In contrast, the concentrations of GIP, PYY and PP were significantly lower after the M-meal in T2D patients (p<0.001). Compared with the V-meal, the M-meal was associated with a larger increase in lipoperoxidation in T2D patients (p<0.05).

**Conclusion/Interpretation:**

Our results suggest that the diet composition and the energy content, rather than the carbohydrate count, should be important considerations for dietary management and demonstrate that processed meat consumption is accompanied by impaired GIH responses and increased oxidative stress marker levels in diabetic patients.

**Trial Registration:**

ClinicalTrials.gov NCT01572402

## Introduction

The current guidelines for the treatment of diabetic patients focus primarily on carbohydrate counts [1]. However, the postprandial metabolic response is likely also modified by the contents of other macronutrients in meals and by gastrointestinal hormone (GIH) release.

Dietary saturated fat is known to impair insulin sensitivity and to enhance hepatic glucose production [2]. Studies of patients suffering from diabetes have revealed that dietary fat delays gastric emptying, leading to a lag in glucose absorption [3], [4]. On the other hand, studies suggest that in patients suffering from diabetes, higher fat meals acutely increase the glucose concentration and the requirement for insulin compared with meals containing similar carbohydrate but lower fat contents [5]. Additionally, addition of protein energy to a meal likely increases the postprandial glucose level [6].

Epidemiological studies suggest a positive association between high consumption of total and red meat and incident T2D [7], [8]. Subjects who consumed any processed meats (salted fish and frankfurters) were 38% more likely to develop diabetes [9]. Previous studies support the concept that increased oxidative stress may play an important role in T2D manifestation [10]. Dietary fat quality has been proposed to be a critical factor. Several studies have suggested that a high intake of saturated fatty acids naturally present in meat contributes to the risk of glucose intolerance [11], [12]. In an intervention study, humans suffering from metabolic syndrome who were consuming a high saturated fatty-acid diet displayed higher oxidative stress markers postprandially [13], [14].

In contrast, some intervention trials demonstrated a greater improvement in insulin sensitivity, glycemic control and a reduction in oxidative stress markers in T2D patients consuming a vegetarian diet compared with those consuming a traditional diabetes diet [15], [16]. The aim of our study was to determine the acute effects of meat and plant-based meals on postprandial GIH secretion. Thus, we designed this randomized crossover study to evaluate the acute (a time span of hours) postprandial response to two standardized meals containing the same caloric content but a different nutritional content: a processed meat (hamburger) meal rich in protein and saturated fat and a plant-based meal rich in carbohydrates. The objective of our study was to examine whether the acute intake of different types of isocaloric meals consumed in amounts typical of normal eating (food intake representative of real life) would be associated with different postprandial changes in glucose, lipid, immunoreactive insulin (IRI), GIH and oxidative stress marker levels in patients with T2D compared with healthy subjects. We investigated the hypothesis that a processed meat meal would lead to an abnormal postprandial increase in the levels of plasma lipids and oxidative stress markers and impaired GIH responses in T2D patients. Our results should contribute knowledge relevant to the dietary management of diabetic patients.

## Methods and Materials

### 1. Study subjects and design

**Figure 1:**
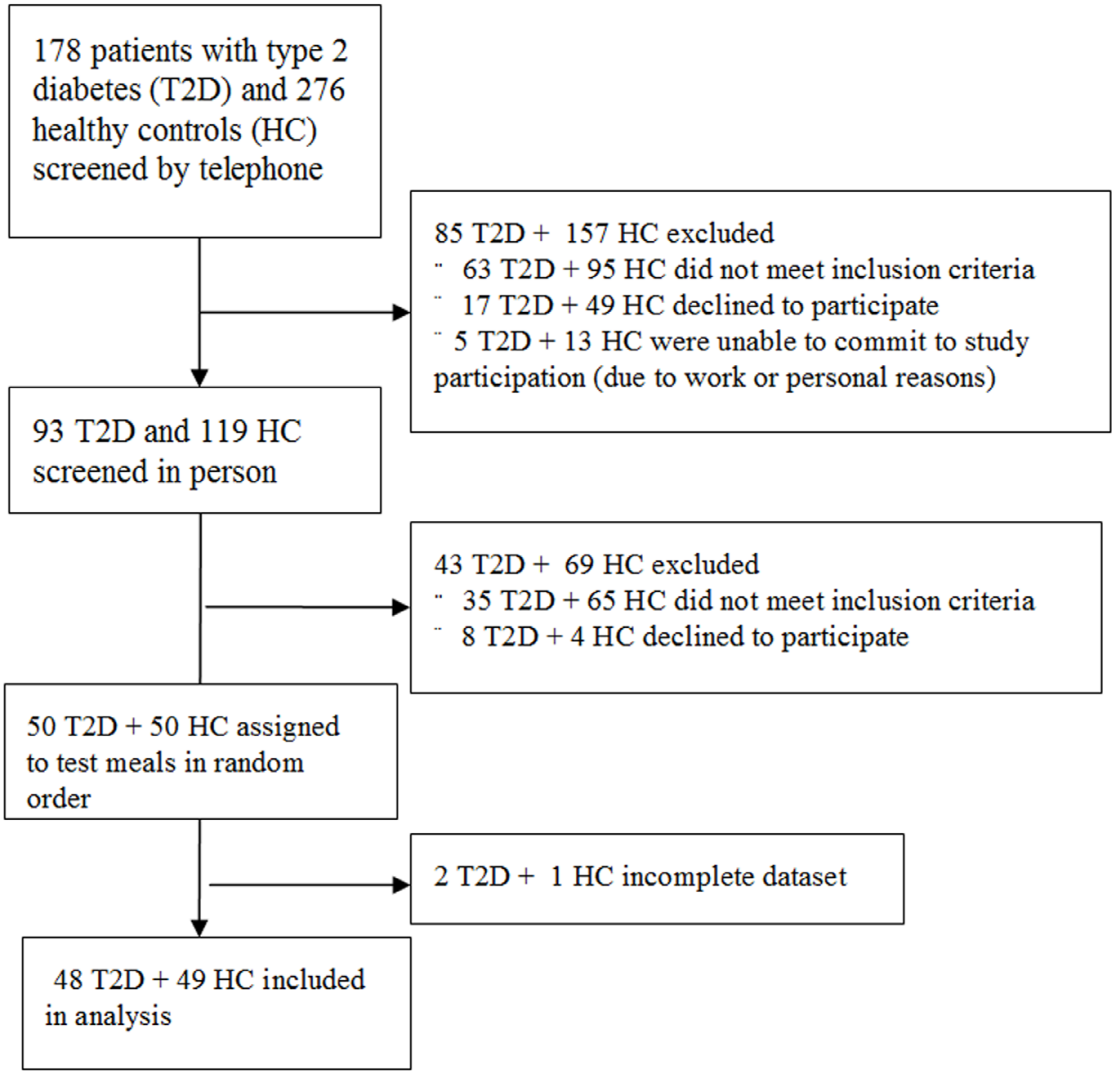
Enrollment of the participants and completion of the study.

The protocol for this trial and supporting CONSORT checklist are available as supporting information; see Checklist S1 and Trial protocol S1 and S2. The study used a prospectively randomized crossover design and included a group of 50 patients suffering from T2D and 50 healthy controls. Their characteristics are presented in Table 1. The mean age was 55; about half of the subjects were men; and the mean duration of diabetes among the T2D patients was 9.8 years. Written informed consent was obtained from all participants prior to enrollment in the study; the study protocol, informed consent, and patient information were reviewed and approved by the Ethics Committee of the Thomayer Hospital and Institute for Clinical and Experimental Medicine in Prague, Czech Republic on November 9, 2011. On January 5, 2012 we started the telephone screening of potential participants. Subjects were screened in person and enrolled into the study between April 3, 2012 and April 20, 2012. The first patient entered the intervention phase on April 10, 2012 and the last completed on May 5, 2012. There was no follow-up phase. The study flowchart is presented in Figure 1. Of the 178 patients with T2D and 276 healthy controls, who were screened, 50 participants of both groups were included and randomized, 2 participants with T2D and 1 healthy subject did not complete the trial. The reasons for study participant exclusion are given in Figure 1. Registration on ClinicalTrials.gov was initiated on April 3, 2012 (Identifier: http://clinicaltrials.gov/show/NCT01572402). The authors confirm that all ongoing and related trials for this intervention are registered.

**Table 1:** General characteristics of the Diabetic and Control Population.

Characteristics	Patients with T2D (n = 50)	Healthy Controls (n = 50)
Age – years	56±6	54±8
Male – No. (%)	23 (46)	23 (46)
Female – No.(%)	27 (54)	27 (54)
Smokers – No. (%)	11 (22)	7 (14)
Weight – kg	96.5±17	71±11
BMI – kg.m^−2^	33.3±5.6	24.4±2.5
Waist – cm	107±13.0	85±8.0
Hips – cm	115±12.0	98±5.0
HbA1c (DCCT) – %	7.0±3.2	5.6±2.4
HbA1c (IFCC) – mmol/mol	53.7±12.0	37.3±2.7
Fasting glucose level – mmol/l	8.0±3.1	5±0.44
Duration of diabetes – years	9.8±6.3	

Data are means ± SD.

### 2. Inclusion and exclusion criteria

The inclusion criteria were men and women aged between 30–70 years with a body mass index (BMI) between 27–50 kg/m^2^. Patients diagnosed with T2D had been treated with diet and/or oral hypoglycemic agents (metformin and/or sulfonylureas) for at least one year. Healthy volunteers exhibiting normal glucose tolerance and without metabolic syndrome or diagnosed diabetes among first-line relatives were included. Subjects exhibiting renal, liver or thyroid disease, drug abuse, including alcoholism, and pregnant or breastfeeding women were excluded.

### 3. Study procedure

All measurements were performed on an outpatient basis after a 10- to 12-h overnight fast with only tap water allowed ad libitum. The diabetic patients did not take any of their diabetes medication the evening or the morning before the assessments. The postprandial state was measured after intake of a standard breakfast – one of two isocaloric test meals in a random order consisting of either a processed meat burger meal (a fastfood burger: cooked-pork seasoned meat in a wheat bun with sesame seeds, tomato, cheddar-type cheese, lettuce, spicy sauce, onion) or a plant-based burger meal (a couscous burger: boiled couscous, baked with onion, garlic, plant oil, spices, oat-flakes in a wheat bun with sesame seeds). For each intervention visit, the meals were delivered fresh from the manufacturer. The study nurses generated the random sequence of the meals and assigned participants to the interventions in one-week intervals. The participants ate the meals under the nurse’s control in the laboratory. Neither the study staff nor the participants were blinded to the content of the meals. The compositions of the meals are presented in Table 2.

**Table 2:** Composition of the test meals.

Meal	Hamburger (M - meal)	Vegan burger (V - meal)
Total weight (g)	150	235
Energy content (kCal/kJ)	455/1904	456/1907
Carbohydrates (g) (%)	31 (27%)	60 (52%)
Proteins (g) (%)	24 (21%)	13 (11%)
Lipids (g) (%)	26 (52%)	19 (37%)
*Saturated fatty acids (g)*	*10*	*6*
*Monounsaturated fatty acids (g)*	*7*	*5*
*Polyunsaturated fatty acids (g)*	*9*	*8*
Fiber (g)	5	8

The plasma concentrations of glucose, immunoreactive insulin, C-peptide, triglycerides (TG), free fatty acids (FFA), oxidative stress markers and GIH were measured during the fasting state (time 0) and then 30, 60, 120 and 180 min after the meal. In this single-center study the samples were collected in the Laboratory of Clinical Pathophysiology in Institute for Clinical and Experimental Medicine.

### 4. Analytic methods

#### 4.1 Metabolic parameters

Plasma glucose was analyzed via the glucose-oxidase method using a Beckman Analyzer (Beckman Instruments Inc., Fullerton, CA, USA). Serum immunoreactive insulin and C-peptide concentrations were determined using Insulin and C-peptide IRMA kits (Immunotech, Prague, Czech Republic), respectively. Plasma lipid concentrations were measured via enzymatic methods (Roche, Basel, Switzerland).

#### 4.2 Gastrointestinal and appetite hormones

Protease and dipeptidyl peptidase-4 inhibitors were added to two samples at each time point. The concentrations of glucagon-like peptide −1 (GLP-1), gastric inhibitory peptide (GIP), pancreatic polypeptide (PP), peptide YY (PYY), leptin and ghrelin were determined via multiplex immunoanalyses based on xMAP technology using a MILLIPLEX MAP Human Gut Hormone Panel (Millipore, Billerica, MA, USA) and a Luminex 100 IS analyzer (Luminex Corporation, Austin, TX, USA) [17]. The assay sensitivities, expressed as the minimum detectable concentrations reported in the instructions for use by the manufacturer, are (in pg/mL): ghrelin 1.8; leptin 157.2; GIP 0.2; GLP-1 5.2; PP 2.4; and PYY 8.4. For our measurements, the sensitivity was set as the value of the lowest standard concentration: ghrelin 13.7; GIP 2.7; GLP-1 13.7; and PP 13.7. For other analytes, there were no values below the corresponding calibration range.

#### 4.3 Oxidative stress markers

The levels of lipid peroxidation were determined via a home-made method using thiobarbituric acid reactive substances (TBARS) [18]. The activity of superoxide dismutase (SOD) was analyzed using a SOD assay kit (Sigma-Aldrich, St Louis, MO, USA). The serum level of ascorbic acid was measured via a home-made method [19]. The level of reduced glutathione in whole blood was determined using a glutathione HPLC diagnostic kit (Chromsystems, Munich, Germany).

### 5. Statistical analyses

For statistical analysis, repeated-measures ANOVA was performed. An estimate of the number of subjects that we recruited was carried out using a power analysis of repeated measurements via PASS 2005 statistical software (Number Cruncher Statistical Systems, Kaysville, UT, USA). The power analysis was completed for the most important variables and the number of subjects should provide the power >0.8 for all these variables. The factors of subject, group, meal and time were included in the analyses. The interactions between meal and time (meal×time) were calculated for each variable. ANOVA was followed by least significant difference (LSD) multiple comparisons post-hoc analysis. The original dependent variable was subjected to a power transformation to obtain constant variance and a symmetric distribution of the data and residuals [20]. The data are presented as the means with 95% CI. We have checked the carry-over effect using the model including the factor “sequence” and we have not found any significance of this factor for any dependent variables.

## Results

### 1. Glucose, C-peptide, insulin and lipid responses

**Figure 2:**
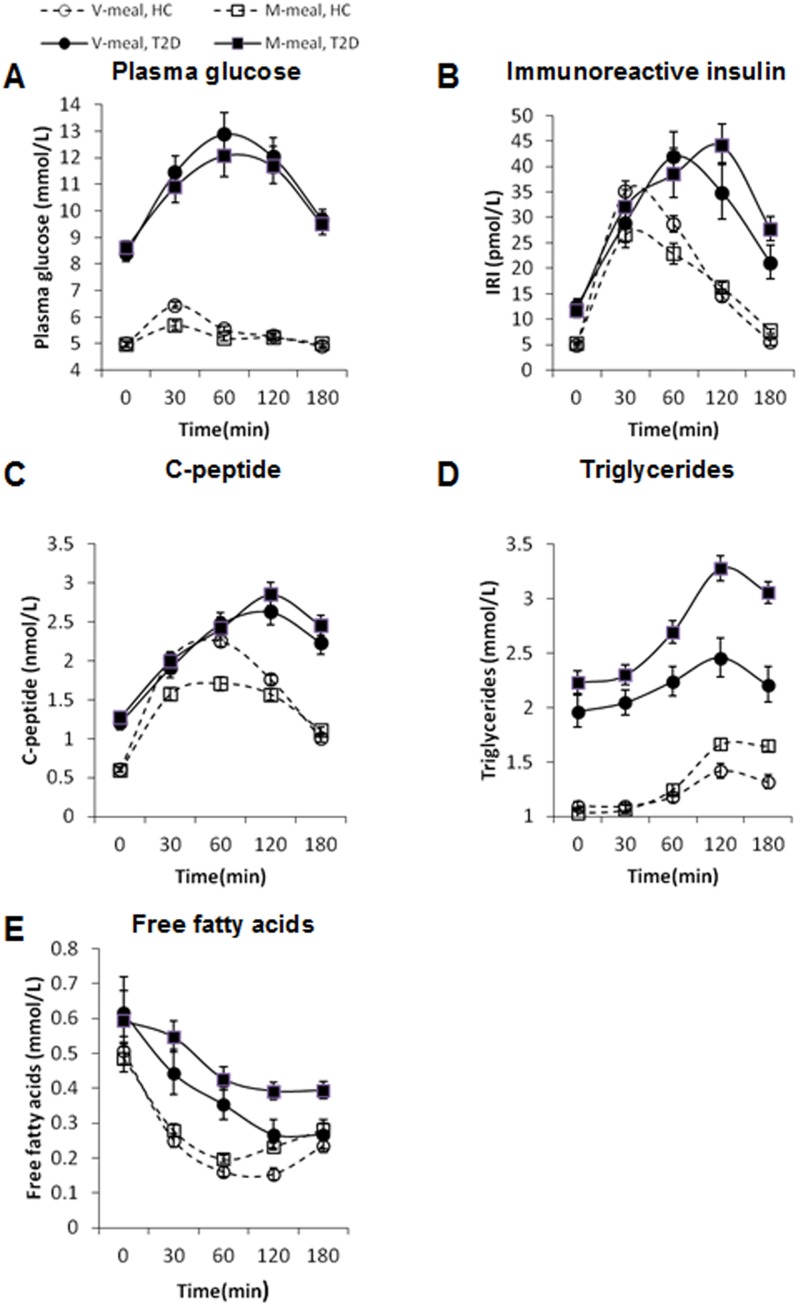
Postprandial changes in plasma concentrations of glucose, immunoreactive insulin, C-peptide, triglycerides and free fatty acids in patients with diabetes (T2D, full line) (n = 48) and healthy controls (HC, dashed line) (n = 49) after ingestion of the V-meal (circles) and the M-meal (squares). Data are expressed as mean with 95%CI. Repeated-measures ANOVA was performed. **A: Plasma glucose: Healthy controls:** Factors diet: p = 0.0001; time: p<0.0001; interaction diet × time: p = 0.0001; **T2D:** diet: p = 0.3767; time: p<0.0001; interaction diet × time: p = 0.708. **B: Immunoreactive insulin: Healthy controls:** Factors diet: p = 0.9156; time: p<0.0001; interaction diet × time: p<0.0001; **T2D:** diet: p = 0.1084; time: p<0.0001; interaction diet × time: p = 0.098. **C:** C-peptide: **Healthy controls:** Factors diet: p<0.0001; time: p<0.0001; interaction diet × time: p<0.0001; **T2D:** diet: p = 0.0713; time: p<0.0001; diet × time: p = 0.6615. **D: Triglycerides: Healthy controls:** Factors diet: p = 0.0001; time: p<0.0001; interaction diet × time: p<0.0001; **T2D:** diet: p<0.0001; time: p<0.0001; interaction diet × time: p = 0.0192. **E: Free fatty acids: Healthy controls:** Factors diet: p<0.0001; time: p<0.0001; interaction diet × time: p = 0.0003; **T2D:** diet: p<0.0001; time: p<0.0001; interaction diet × time: p = 0.058.

The plasma concentrations of glucose, IRI, C-peptide and lipids during the fasting state and their postprandial profiles are illustrated in Fig. 2. All of these measured parameters were significantly higher in the T2D patients than in healthy controls at nearly every time point. The fasting concentrations did not differ before the M- and V-meals in both groups. In both the diabetic and healthy subjects, the postprandial plasma levels of glucose were significantly higher after the V-meal at only one time point – at peak blood glucose level, after 30 min in healthy subjects and 60 min in patients with T2D. The two different meals induced relatively similar glucose responses in both groups when the time-course is considered. Although the M-meal resulted in a significantly lower IRI and C-peptide response than the V-meal in healthy subjects, these levels decreased more slowly, and after 180 min the IRI after the M-meal was significantly higher in both groups of subjects. In the T2D patients, the postprandial increases in IRI and C-peptide were not significantly different after both diets, and the peak of the IRI curve was considerably postponed. After the M-meal, hyperinsulinemia persisted longer in both groups. The plasma concentration of triglycerides inversely correlated to the plasma concentration of free fatty acids. The M-meal resulted in a significantly higher postprandial increase in triglycerides and a further decrease in free fatty acids in both groups, and these differences were more pronounced among the patients with T2D.

### 2. GIHs

**Figure 3:**
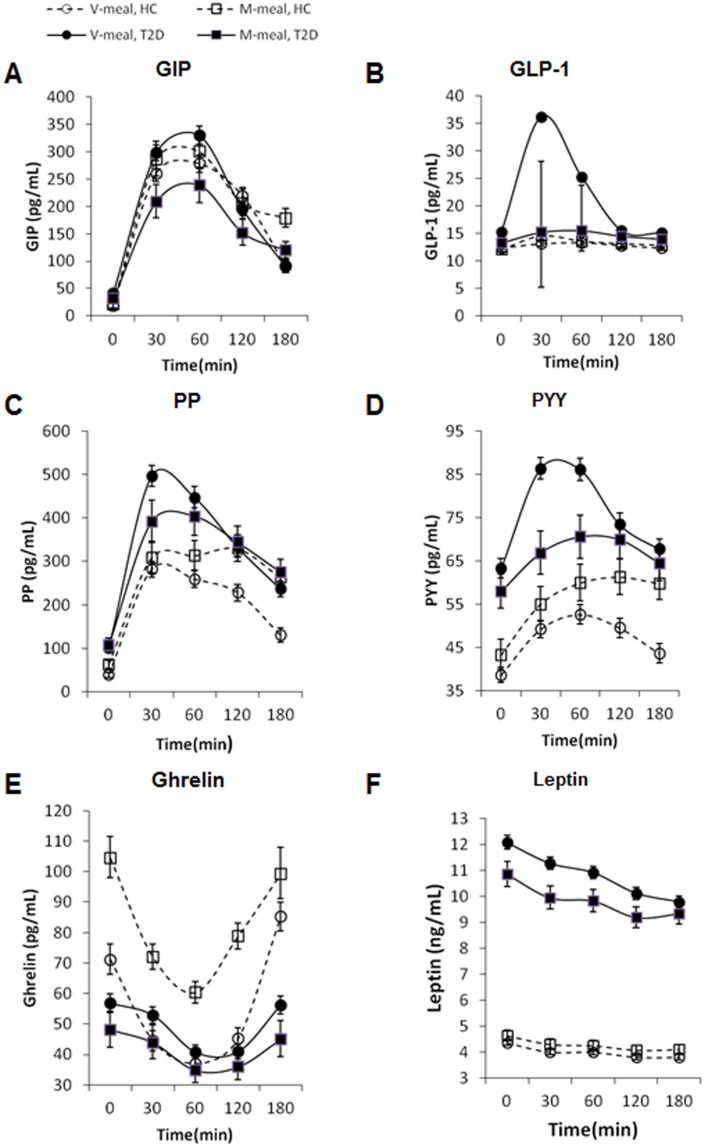
Postprandial changes in plasma concentrations of gastrointestinal and appetite hormones in patients with diabetes (T2D, full line) (n = 48) and healthy controls (HC, dashed line) (n = 49) after ingestion of the V-meal (circles) and the M-meal (squares). Data are expressed as mean with 95%CI. Repeated-measures ANOVA was performed. **A: GLP-1: Healthy controls:** Factors diet: p = 0.1072; time: p<0.0001; interaction diet × time: p = 0.2731; **T2D:** diet: p<0.0001; time: p<0.0001; interaction diet × time: p<0.0001. **B: GIP: Healthy controls:** Factors diet: p = 0.0128; time: p<0.0001; interaction diet × time: p = 0.0001; **T2D:** diet: p = 0.0005; time: p<0.0001; interaction diet × time: p<0.0001. **C: PP: Healthy controls:** Factors diet: p<0.0001; time: p<0.0001; interaction diet × time: p = 0.0016; **T2D:** diet: p = 0.5702; time: p<0.0001; interaction diet × time: p = 0.0124. **D: PYY: Healthy controls:** Factors diet: p<0.0001; time: p<0.0001; interaction diet × time: p = 0.0768; **T2D:** diet: p<0.0001; time: p<0.0001; interaction diet × time: p = 0.004. **E: Ghrelin: Healthy controls:** Factors diet: p<0.0001; time: p<0.0001; interaction diet × time: p = 0.0004; **T2D:** diet: p = 0.0002; time: p<0.0001; interaction diet × time: p = 0.9519. **F: Leptin: Healthy controls:** Factors diet: p = 0.0019; time: p<0.0001; intraction diet × time: p = 0.9983; **T2D:** diet: p<0.0001; time: p<0.0001; interaction diet × time: p = 0.4988.

The basal concentrations of nearly all of the GIHs were significantly increased in the T2D patients compared with the healthy controls. There was a considerable difference in the GIH response after ingestion of the M-meal compared with the V-meal in both healthy subjects and the T2D patients (Fig. 3). The V-meal resulted in a significantly higher GIH response than the M-meal but only among the T2D patients. In healthy subjects, the postprandial GIH levels were significantly lower after the V-meal than after the M-meal. The largest difference was detected in the postprandial concentrations of PP and PYY. In the T2D patients, the postprandial concentrations of GLP-1, GIP and PYY were significantly higher after the V-meal. The largest difference between the two meals was detected in the postprandial secretion of GLP-1, both with respect to quantity and dynamics.

### 3. Appetite hormones

The concentrations of ghrelin and leptin differed significantly between the T2D patients and the healthy controls throughout the entire meal assessment (Fig. 3). In the fasting state, the plasma concentration of ghrelin was 56% lower in the diabetic subjects (p<0.001), and the plasma concentration of leptin was 150% higher than the healthy controls (p<0.001). The plasma concentration of ghrelin was significantly lower and that of leptin was significantly higher in the T2D patients throughout the entire meal assessment, with no difference between the two isocaloric diets. The physiological postprandial suppression of ghrelin secretion was less pronounced in the diabetic subjects than in the healthy controls, and there was a rapid decrease in the ghrelin level beyond the first 60 min after consumption, and the postprandial decrease was significantly larger after the M-meal among healthy controls (p<0.001).

We have added a subanalysis by adding a “baseline ghrelin” factor to the model. The baseline ghrelin was the most important factor when included in the model with F = 187, p<0.0001. It means that the higher the baseline level of ghrelin is, the larger the drop. The difference between the two meals remained significant with F = 19.43, p<0.001.

### 4. Oxidative stress parameters

**Figure 4:**
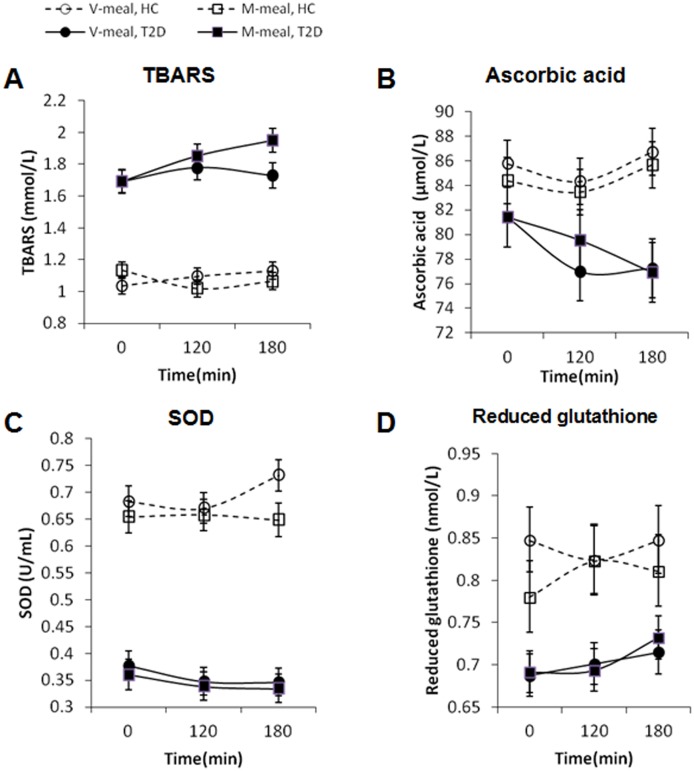
Postprandial changes in plasma concentrations of oxidative stress markers in patients with diabetes (T2D, full line) (n = 45) and healthy controls (HC, dashed line) (n = 49) after ingestion of the V-meal (circles) and the M-meal (squares). Data are expressed as mean with 95% CI. Repeated-measures ANOVA was performed. **A: TBARS: Healthy controls:** Factors diet: p = 0.5952; time: p = 0.5647; interaction diet × time: p = 0.0405; **T2D:** diet: p = 0.0317; time: p = 0.0138; interaction diet × time: p = 0.113. **B: Ascorbic acid:** **Healthy controls:** Factors diet: p = 0.3292; time: p = 0.2324; interaction diet × time: p = 0.9802; **T2D:** diet: p = 0.6016; time: p = 0.0378; interaction diet × time: p = 0.6626. **C:** **Superoxide dismutase**: **Healthy controls:** Factors diet: p = 0.0171; time: p = 0.4608; interaction diet × time: p = 0.2388; **T2D:** diet: p = 0.4312; time: p = 0.2792; interaction diet × time: p = 0.9834. **D: Reduced glutathione: Healthy controls:** Factors diet: p = 0.1504; time: p = 0.8466; interaction diet × time: p = 0.5018; **T2D:** diet: p = 0.7654; time: p = 0.1475; interaction diet × time: p = 0.7935.

The fasting concentrations of all of the oxidative stress markers differed between the groups. (Fig. 4). In the T2D patients, compared with the control subjects, the levels of TBARS, a measure of lipoperoxidation, were increased by 67% (p<0.001), while the levels of ascorbic acid, reduced glutathione and SOD activity were decreased by 5%, 13% and 48%, respectively (p<0.001).

During the postprandial phase, consumption of the M-meal was associated with a significantly greater increase in the TBARS levels in diabetic patients than that of the V-meal. In the healthy controls, no postprandial dynamics of the TBARS levels were detected. The SOD activity in the healthy controls was significantly increased after the V-meal compared with the M-meal. There was no significant change in the plasma concentration of SOD during the postprandial phase in diabetic subjects after either meal. The plasma concentrations of reduced glutathione and ascorbic acid did not change significantly in either patients with diabetes or healthy subjects after either meal.

## Discussion

### 1. Postprandial changes in the plasma concentrations of glucose, lipids and insulin

Our results suggest that after two isocaloric meals containing different carbohydrate contents (M- and V-meals), the postprandial plasma glucose levels during the first 180 min after meal ingestion differed significantly only with respect to the peak glucose levels. Some human studies suggest that other macronutrients can modify the postprandial insulin demand and the glycemic response and therefore exert an additional impact on the postprandial increase in the glucose levels, as shown in healthy subjects [21] as well as patients suffering from T1D [6] or T2D [22].

Concerning the lipid levels, the significantly higher postprandial increase in the triglyceride levels and the smaller decrease in the free fatty acid levels detected in both groups after the M-meal is not surprising. Free fatty acids are known to impair insulin sensitivity and to enhance hepatic glucose production [2]. Decreases in the levels of these components leads to improvements in insulin sensitivity and glucose tolerance based on intervention studies [23].

We initially detected significantly lower IRI and C-peptide responses after the M-meal than the V-meal in healthy subjects. However, these levels decreasing more slowly, and after 180 min, the level of IRI was significantly higher in both groups after the M-meal. This result indicates that after the M-meal, hyperinsulinemia persisted longer in both groups.

This finding is in accordance with a glucose clamp study of non-diabetic human subjects indicating that increases in the FFA levels lead to insulin resistance within several hours [24]. Several studies confirmed that meals containing higher protein and/or fat contents exert an additional impact on the postprandial insulin demand and the increase in the glucose levels. In patients suffering from type 1 diabetes, high-fat meals containing identical carbohydrate and protein contents required a larger dose of insulin than low-fat meals [5]. Furthermore, addition of protein increases the insulin demand [6].

### 2. Postprandial changes in the plasma concentrations of GIHs and appetite hormones

Carbohydrate, fat and protein in the lumen of the gut have been demonstrated to stimulate the secretion of a broad range of GIHs [25].

The incretin effect, the postprandial augmentation of insulin secretion by gut hormones, has been primarily associated with the secretion and insulinotropic effect of two GIHs – GIP and GLP-1. The incretin effect is thought to mediate approximately 50–70% of the overall insulin response after a mixed meal or glucose ingestion in healthy subjects [26], [27].

It is well understood that in patients with diabetes, the incretin effect is diminished secondarily [28], [29], and this result appears to be due to impaired beta cell sensitivity [30].

The mechanisms underlying the loss of incretin activity remain incompletely understood, but several hypotheses have been proposed, including hyperglycemia- and hyperlipidemia-associated receptor desensitization [31], [32].

Studies that have examined the GIP and GLP-1 responses after meals differing with respect to caloric and macronutrient content report inconsistent results regarding the contribution of meal composition to the stimulation of these hormones. One study reported a higher GIP response after a mixed meal than after an oral glucose load [33]. Another study demonstrated that fat preferentially stimulated GIP secretion, whereas carbohydrates stimulated GLP-1 secretion, both regardless of the diabetes status [34]. Dietary protein exerted a variable effect on incretin release [35], [36].

Our results suggest that the intake of each type of isocaloric meal consumed in amounts typical of normal eating was associated with different postprandial changes in the GIP and GLP-1 plasma levels. In the T2D patients, the V-meal resulted in a larger release of GLP-1 and GIP than the M-meal. This result is in accordance with a study suggesting that the GLP-1 response after fat intake is impaired in T2D patients [37]. In contrast, in healthy individuals, the GLP-1 and GIP responses after ingestion of the V-meal were smaller than after the M-meal.

Our data suggest that carbohydrates appear to represent the strongest activator of GLP-1 and GIP secretion among T2D patients, whereas healthy subjects display higher GLP-1, GIP, PYY and PP responses to the mixed meal containing higher fat and protein contents.

In our study, we demonstrated increased basal and postprandial levels of GIP and GLP-1 in the T2D patients compared with the healthy subjects. This finding is in accordance with a study that evaluated the GLP-1 secretion levels and indicated relatively unchanged or even elevated secretion in response to oral nutrition intake in subjects suffering from diabetes [38].

Additionally, a recent meta-analysis revealed that T2D patients do not exhibit reduced GLP-1 secretion in response to an OGTT or a meal test [39]. With respect to GIP secretion, most studies quantifying GIP secretion reported that the GIP levels are normal or even higher in T2D subjects compared with healthy controls [40]. However, reductions in the GLP-1 levels were found in patients suffering from a long disease duration and poor compensation [41]. A recent meta-analysis revealed a negative influence of HbA1c levels on plasma GLP-1 responses [39].

Other gastrointestinal peptides play a role in the regulation of energy intake, appetite and overall energy homeostasis in humans. They are secreted by different cells in the intestine and from the pancreas, and their release is modulated by food ingestion.

PYY is co-secreted predominantly from the endocrine L cells in the ileum together with GLP-1. PYY plays an important regulatory role in GIT function [42].

PP is secreted from endocrine cells in the pancreas. Both PYY and PP, when infused intravenously, reduce appetite and energy intake in healthy humans [43]. They are secreted in response to all three macronutrients, with fat being the most potent stimulus. In healthy humans, it was shown that the presence of fat in the small intestine induced stimulation of PYY and PP in a manner dependent on fat digestion and the presence of free fatty acids [44]. This finding is in accordance with our results; in healthy subjects, we detected significantly greater secretion of PYY and PP after ingestion of the M-meal compared with the V-meal.

The course of PYY secretion consisted of two phases. The initial rise in the PYY level is likely due to a link to the proximal small intestine, and the continuous rise reflects the direct contact of lipids with the distal small intestine [45]. This result was not detected in our diabetic group of patients; their postprandial response with respect to both peptides was significantly higher after the V-meal. Similarly, a previous study suggested that both insulin resistance and abnormal glucose metabolism impaired the PYY response to fat intake [37]. To the best of our knowledge, this is the first report of the different secretory responses of PP after both isocaloric meals in T2D patients compared with healthy controls.

Ghrelin stimulates appetite and food intake and is secreted in the stomach. In healthy individuals, the ghrelin plasma concentrations increase during fasting and are suppressed by meal intake [46], likely via postprandial hyperinsulinemia [47], [48].

We detected postprandial suppression of ghrelin secretion during the first 60 min after meal ingestion. The postprandial decrease depended significantly on the baseline level of ghrelin, which means that the higher the baseline level of ghrelin is, the larger the drop. Furthermore, it was significantly greater after the M-meal in healthy subjects. This finding is in accordance with a study that demonstrates that fat digestion is required for the suppression of ghrelin [44]. The related changes in ghrelin could be deemed positive with respect to the meat meal. We detected lower fasting and postprandial plasma concentrations of ghrelin and a diminished postprandial suppression of ghrelin secretion in the T2D patients, with no difference between the two meals in time.

### 3. Postprandial changes in the plasma concentrations of oxidative stress markers

The postprandial state, characterized by hyperglycemia and hyperlipidemia, is associated with increased oxidative stress in patients suffering from diabetes [49]. In our group of T2D patients, we detected significantly higher fasting concentrations of all oxidative stress markers and significantly lower concentrations of blood antioxidants. Previous studies support the concept that increased oxidative stress may play an important role in T2D manifestation [10]. Dietary fat quality has been proposed to be a critical factor. Several studies have suggested that a high intake of saturated fatty acids naturally present in meat contributes to the risk of glucose intolerance [11], [12]. Based on an intervention study, humans suffering from metabolic syndrome who were consuming a high saturated fatty acid diet displayed higher levels of oxidative stress markers postprandially [13], [14]. We found that in diabetic patients during the postprandial phase, the processed meat meal was associated with a significantly greater increase in the products of oxidative damage, namely TBARS, compared with the plant based meal. This finding could also be due to the method of cooking at a high temperature. It has been reported that most of the lipid-peroxidation products ingested from popular foods are derived from meat products and high-fat processed foods such as red meats cooked at high temperatures [50]. Another study has shown that adding antioxidants to hamburger meat could significantly reduce the formation of lipid-peroxidation products during cooking [51].

### 4. Strengths and limitations of the study

The strength of our study was that we took a physiological approach to measure the postprandial state. We selected two simple isocaloric meals at amounts corresponding to those ingested during a typical meal. The M-meal was rich in both saturated fat and protein; thus, we cannot separate the effect of each macronutrient.

This study also had several limitations. First, the T2D patients exhibited a significantly higher body weight and BMI compared with the control subjects, and these differences may have affected some of the responses reported. The object of our study was to compare the effect of the two different meals within each group (patients with T2D and healthy subjects). In healthy subjects, no effect of adiposity on the postprandial GIP and GLP-1 levels has been reported [52]. The effect of obesity on other GIHs is unclear. In this study, we primarily aimed to compare the two meals rather than the two groups of subjects.

Finally, the 3-h study duration might be insufficient for complete absorption of the nutrients, and this limitation may have led to an underestimation of the measured parameters.

## Conclusions

In conclusion, according to our hypothesis, our results suggest that a processed meat meal is accompanied by an impaired GIH response and increased oxidative stress marker levels in diabetic patients. Our results revealed comparable glycemic responses to isocaloric meals containing different carbohydrate contents, despite the distinct peak glucose concentrations. This finding illustrates that the diet composition and the energy content, rather than the carbohydrate count or the glycemic load, should be important considerations for the dietary management of diabetes.

## Supporting Information

Checklist S1 **CONSORT Checklist.**
(DOC)

Protocol S1 **Trial Protocol in English.**
(DOC)

Protocol S2 **Trial Protocol in Czech.**
(DOC)

## References

[1] American Diabetes Association (2013) Standards of medical care in diabetes–2013. Diabetes Care 36 Suppl 1S11–66. 23264422 10.2337/dc13-S011 PMC3537269

[2] Savage DB, Petersen KF, Shulman GI (2007) Disordered lipid metabolism and the pathogenesis of insulin resistance. Physiol Rev 87: 507–20. 17429039 10.1152/physrev.00024.2006 PMC2995548

[3] Lodefalk M, Aman J, Bang P (2008) Effects of fat supplementation on glycaemic response and gastric emptying in adolescents with Type 1 diabetes. Diabet Med: 1030–5. doi:10.1111/j.1464-5491.2008.02530. 10.1111/j.1464-5491.2008.02530.x 19183308

[4] Gentilcore D, Chaikomin R, Jones KL, Russo A, Feinle-Bisset C, et al (2006) Effects of fat on gastric emptying of and the glycemic, insulin, and incretin responses to a carbohydrate meal in type 2 diabetes. J Clin Endocrinol Metab 91: 2062–7. 16537685 10.1210/jc.2005-2644

[5] Wolpert HA, Atakov-Castillo A, Smith SA, Steil GM (2013) Dietary fat acutely increases glucose concentrations and insulin requirements in patients with type 1 diabetes: implications for carbohydrate-based bolus dose calculation and intensive diabetes management. Diabetes Care 36: 810–6. 23193216 10.2337/dc12-0092 PMC3609492

[6] Peters AL, Davidson MB (1993) Protein and fat effects on glucose responses and insulin requirements in subjects with insulin-dependent diabetes mellitus. Am J Clin Nutr 58: 555–60. 8379513 10.1093/ajcn/58.4.555

[7] Aune D, Ursin G, Veierød MB (2009) Meat consumption and the risk of type 2 diabetes: a systematic review and meta-analysis of cohort studies. Diabetologia 52: 2277–87. 19662376 10.1007/s00125-009-1481-x

[8] Pan A1, Sun Q, Bernstein AM, Schulze MB, Manson JE, et al (2011) Red meat consumption and risk of type 2 diabetes: 3 cohorts of US adults and an updated meta-analysis. Am J Clin Nutr 94: 1088–96. 21831992 10.3945/ajcn.111.018978 PMC3173026

[9] Pan A1, Sun Q, Bernstein AM, Schulze MB, Manson JE, et al (2008) Meats, processed meats, obesity, weight gain and occurrence of diabetes among adults: findings from Adventist Health Studies. Ann Nutr Metab 52: 96–104. 18349528 10.1159/000121365

[10] Ceriello A, Motz E (2004) Is oxidative stress the pathogenic mechanism underlying insulin resistance, diabetes, and cardiovascular disease? The common soil hypothesis revisited. Arterioscler Thromb Vasc Biol 24: 816–23. 14976002 10.1161/01.ATV.0000122852.22604.78

[11] Feskens EJ, Virtanen SM, Räsänen L, Tuomilehto J, Stengård J, et al (1995) Dietary factors determining diabetes and impaired glucose tolerance. A 20-year follow-up of the Finnish and Dutch cohorts of the Seven Countries Study. Diabetes Care 18: 1104–12. 7587845 10.2337/diacare.18.8.1104

[12] Maron DJ, Fair JM, Haskell WL (1991) Saturated fat intake and insulin resistance in men with coronary artery disease. The Stanford Coronary Risk Intervention Project Investigators and Staff Circulation 84: 2020–7. 1934376 10.1161/01.cir.84.5.2020

[13] Perez-Martinez P1, Garcia-Quintana JM, Yubero-Serrano EM, Tasset-Cuevas I, Tunez I, et al (2010) Postprandial oxidative stress is modified by dietary fat: evidence from a human intervention study. Clin Sci (Lond) 15: 251–61. 10.1042/CS20100015 20420579

[14] Cardona F, Túnez I, Tasset I, Montilla P, Collantes E, et al (2008) Fat overload aggravates oxidative stress in patients with the metabolic syndrome. Eur J Clin Invest 38: 510–5. 18489583 10.1111/j.1365-2362.2008.01959.x

[15] Barnard ND1, Katcher HI, Jenkins DJ, Cohen J, Turner-McGrievy G (2009) Vegetarian and vegan diets in type 2 diabetes management. Nutr Rev 67: 255–63. 19386029 10.1111/j.1753-4887.2009.00198.x

[16] Kahleova H, Matoulek M, Malinska H, Oliyarnik O, Kazdova L, et al (2011) Vegetarian diet improves insulin resistance and oxidative stress markers more than conventional diet in subjects with Type 2 diabetes. Diabet Med 28: 549–59. 21480966 10.1111/j.1464-5491.2010.03209.x PMC3427880

[17] Kellar KL, Iannone MA (2002) Multiplexed microsphere-based flow cytometric assays. Exp Hematol 30: 1227–37. 12423675 10.1016/s0301-472x(02)00922-0

[18] Yokode M1, Kita T, Kikawa Y, Ogorochi T, Narumiya S, et al (1988) Stimulated arachidonate metabolism during foam cell transformation of mouse peritoneal macrophages with oxidized low density lipoprotein. J. Clin Invest 81: 720–9. 3125226 10.1172/JCI113377 PMC442519

[19] Nakagawa K, Kanno H, Miura Y (1997) Detection and analyses of ascorbyl radical in cerebrospinal fluid and serum of acute lymphoblastic leukemia. Anal Biochem 254: 31–5. 9398342 10.1006/abio.1997.2372

[20] Meloun M, Hill M, Militký J, Kupka K (2000) Transformation in the PC-aided biochemical data analysis. Clin Chem Lab Med 38: 553–9. 10987205 10.1515/CCLM.2000.081

[21] Nuttall FQ, Gannon MC, Wald JL, Ahmed M (1985) Plasma glucose and insulin profiles in normal subjects ingesting diets of varying carbohydrate, fat and protein content. J Am Coll Nutr 4: 437–50. 3900180 10.1080/07315724.1985.10720086

[22] Estrich D, Ravnik A, Schlierf G, Fukayama G, Kinsell L, et al (1967) Effects of Co-ingestion of fat and protein upon carbohydrate-induced hyperglycemia. Diabetes 16: 232–7. 6023165 10.2337/diab.16.4.232

[23] Bajaj M, Suraamornkul S, Kashyap S, Cusi K, Mandarino L, et al (2004) Sustained reduction in plasma free fatty acid concentration improves insulin action without altering plasma adipocytokine levels in subjects with strong family history of type 2 diabetes. J Clin Endocrinol Metab 89: 4649–55. 15356076 10.1210/jc.2004-0224

[24] Gormsen LC, Nielsen C, Jessen N, Jørgensen JO, Møller N (2011) Time-course effects of physiological free fatty acid surges on insulin sensitivity in humans. Acta Physiol 201: 349–56. 10.1111/j.1748-1716.2010.02181.x 20731625

[25] Tolhurst G, Reimann F, Gribble FM (2012) Intestinal sensing of nutrients. Handb Exp Pharmacol 209: 309–35. 10.1007/978-3-642-24716-3_14 22249821

[26] Creutzfeld W (1979) The incretin concept today. Diabetologia 16: 75–85. 32119 10.1007/BF01225454

[27] Nauck MA, Homberger E, Siegel EG, Allen RC, Eaton RP, et al (1986) Incretin effects of increasing glucose loads in man calculated from venous insulin and C-peptide responses. J Clin Endocrinol Metab 63: 492–8. 3522621 10.1210/jcem-63-2-492

[28] Nauck M, Stöckmann F, Ebert R, Creutzfeldt W, et al (1986) Reduced incretin effect in type 2 (non-insulin-dependent) diabetes. Diabetologia 29: 46–52. 3514343 10.1007/BF02427280

[29] Knop FK, Vilsbøll T, Højberg PV, Larsen S, Madsbad S, et al (2007) Reduced incretin effect in type 2 diabetes: cause or consequence of the diabetic state? Diabetes 56: 1951–9. 17513701 10.2337/db07-0100

[30] Kjems LL, Holst JJ, Vølund A, Madsbad S (2003) The influence of GLP-1 on glucose-stimulated insulin secretion: effects on beta-cell sensitivity in type 2 and nondiabetic subjects. Diabetes 52: 380–6. 12540611 10.2337/diabetes.52.2.380

[31] Xu G1, Kaneto H, Laybutt DR, Duvivier-Kali VF, Trivedi N, et al (2007) Downregulation of GLP-1 and GIP receptor expression by hyperglycemia: possible contribution to impaired incretin effects in diabetes. Diabetes 56: 1551–8. 17360984 10.2337/db06-1033

[32] Kang ZF1, Deng Y, Zhou Y, Fan RR, Chan JC, et al (2013) Pharmacological reduction of NEFA restores the efficacy of incretin-based therapies through GLP-1 receptor signalling in the beta cell in mouse models of diabetes. Diabetologia 56: 423–33. 23188390 10.1007/s00125-012-2776-x PMC3536946

[33] Vollmer K1, Holst JJ, Baller B, Ellrichmann M, Nauck MA, et al (2008) Predictors of incretin concentrations in subjects with normal, impaired and diabetic glucose tolerance. Diabetes 57: 678–87. 18057091 10.2337/db07-1124

[34] Rijkelijkhuizen JM1, McQuarrie K, Girman CJ, Stein PP, Mari A, et al (2010) Effects of meal size and composition on incretin, alpha-cell, and beta-cell responses. Metabolism 59: 502–11. 19846181 10.1016/j.metabol.2009.07.039

[35] Carr RD1, Larsen MO, Winzell MS, Jelic K, Lindgren O, et al (2008) Incretin and islet hormonal responses to fat and protein ingestion in healthy men. Am J Physiol Endocrinol Metab 295: E779–84. 18612044 10.1152/ajpendo.90233.2008

[36] Carrel G1, Egli L, Tran C, Schneiter P, Giusti V, et al (2011) Contributions of fat and protein to the incretin effect of a mixed meal. Am J Clin Nutr 94: 997–1003. 21849595 10.3945/ajcn.111.017574 PMC3742299

[37] Fernández-García JC1, Murri M, Coin-Aragüez L, Alcaide J, El Bekay R, et al (2014) GLP-1 and peptide YY secretory response after fat load is impaired by insulin resistance, impaired fasting glucose and type 2 diabetes in morbidly obese subjects. Clin Endocrinol (Oxf) 80: 671–6. 23573808 10.1111/cen.12221

[38] Meier JJ, Nauck MA (2010) Is the diminished incretin effect in type 2 diabetes just an epi-phenomenon of impaired beta-cell function? Diabetes 59: 1117–25. 20427697 10.2337/db09-1899 PMC2857890

[39] Calanna S1, Christensen M, Holst JJ, Laferrère B, Gluud LL, et al (2013) Secretion of glucagon-like peptide-1 in patients with type 2 diabetes mellitus: systematic review and meta-analyses of clinical studies. Diabetologia 56: 965–72. 23377698 10.1007/s00125-013-2841-0 PMC3687347

[40] Cernea S (2011) The role of incretin therapy at different stages of diabetes. Rev Diabet Stud Fall 8: 323–38. 10.1900/RDS.2011.8.323 PMC3280667 22262070

[41] Vilsbøll T, Krarup T, Deacon CF, Madsbad S, Holst JJ (2001) Reduced postprandial concentrations of intact biologically active glucagon-like peptide 1 in type 2 diabetic patients. Diabetes 50: 609–13. 11246881 10.2337/diabetes.50.3.609

[42] Kim BJ1, Carlson OD, Jang HJ, Elahi D, Berry C, et al (2005) Peptide YY is secreted after oral glucose administration in a gender-specific manner. J Clin Endocrinol Metab 90: 6665–71. 16174724 10.1210/jc.2005-0409

[43] Batterham RL1, Le Roux CW, Cohen MA, Park AJ, Ellis SM, et al (2003) Pancreatic polypeptide reduces appetite and food intake in humans. J Clin Endocrinol Metab 88: 3989–92. 12915697 10.1210/jc.2003-030630

[44] Feinle-Bisset C1, Patterson M, Ghatei MA, Bloom SR, Horowitz M (2005) Fat digestion is required for suppression of ghrelin and stimulation of peptide YY and pancreatic polypeptide secretion by intraduodenal lipid. Am J Physiol Endocrinol Metab 289: E948–53. 15998659 10.1152/ajpendo.00220.2005

[45] Adrian TE, Ferri GL, Bacarese-Hamilton AJ, Fuessl HS, Polak JM, et al (1985) Human distribution and release of a putative new gut hormone, peptide YY. Gastroenterology 89: 1070–7. 3840109 10.1016/0016-5085(85)90211-2

[46] Wren AM1, Small CJ, Ward HL, Murphy KG, Dakin CL, et al (2000) The novel hypothalamic peptide ghrelin stimulates food intake and growth hormone secretion. Endocrinology 141: 4325–8. 11089570 10.1210/endo.141.11.7873

[47] Blom WA, Stafleu A, de Graaf C, Kok FJ, Schaafsma G, et al (2005) Ghrelin response to carbohydrate-enriched breakfast is related to insulin. Am J Clin Nutr 81: 367–75. 15699223 10.1093/ajcn.81.2.367

[48] Hagemann D1, Holst JJ, Gethmann A, Banasch M, Schmidt WE, et al (2007) Glucagon-like peptide 1 (GLP-1) suppresses ghrelin levels in humans via increased insulin secretion. Regul Pept 143: 64–8. 17434608 10.1016/j.regpep.2007.03.002

[49] Ceriello A, Esposito K, Testa R, Bonfigli AR, Marra M, et al (2011) The possible protective role of glucagon-like peptide 1 on endothelium during the meal and evidence for an “endothelial resistance” to glucagon-like peptide 1 in diabetes. Diabetes Care 34: 697–702. 21273492 10.2337/dc10-1949 PMC3041210

[50] Goldberg T1, Cai W, Peppa M, Dardaine V, Baliga BS, et al (2004) Advanced glycoxidation end products in commonly consumed foods. J Am Diet Assoc 104: 1287–91. 15281050 10.1016/j.jada.2004.05.214

[51] Li Z1, Henning SM, Zhang Y, Zerlin A, Li L, et al (2010) Antioxidant-rich spice added to hamburger meat during cooking results in reduced meat, plasma, and urine malondialdehyde concentrations. Am J Clin Nutr 91: 1180–4. 20335545 10.3945/ajcn.2009.28526 PMC2854897

[52] Runchey SS, Valsta LM, Schwarz Y, Wang C, Song X, et al (2013) Effect of low- and high-glycemic load on circulating incretins in a randomized clinical trial. Metabolism 62: 188–95. 22959497 10.1016/j.metabol.2012.07.006 PMC3519963

