# Supplementary material for: Differential Acute Postprandial Effects of Processed Meat and Isocaloric Vegan Meals on the Gastrointestinal Hormone Response in Subjects Suffering from Type 2 Diabetes and Healthy Controls: A Randomized Crossover Study
Source: PLoS One. 2014 Sep 15;9(9):e107561. doi: 10.1371/journal.pone.0107561 (PMC4164634; doi:10.1371/journal.pone.0107561)
Supplement: Protocol S2 — Trial Protocol in Czech. (DOC) [file pone.0107561.s003.doc]

**Protokol**

**Vliv tří jídel se stejným kalorickým obsahem ale různým složením na sekreci inzulínu a peptidů ze zažívacího traktu u nemocných s diabetem 2. typu a u zdravých kontrol**

Cukrovka 2. typu je charakterizována sníženou sekrecí inkretinů, hormonů produkovaných v zažívacím traktu, které mají vliv na sekreci inzulínu. V tomto našm výzkumném projektu chceme zkoumat vliv tří jídel se stejným kalorickým obsahem ale různým složením na sekreci inzulínu a peptidů ze zažívacího traktu u nemocných s diabetem 2. typu a u zdravých kontrol.

Účastníci studie budou absolvovat 3 křivky po jídle:

1. po bagetě Crocodille Sýrový mlsoun
2. po kuskusovém sendviči Countrylife
3. po McCountry sendviči od McDonalda

**Každá křivka po jídle** bude trvat 180 min. Na tuto návštěvu účastníci studie přijdou v 7-8 h nalačno (po 8-12 h lačnění), večer a ráno před vyšetřením nebudou pacienti s cukrovkou brát léky na cukrovku.

**Cíl:** Porovnat glykemie, sekreci inzulínu a peptidů z gastrointestinálního traktu u 60 nemocných s diabetem 2. typu a u 60 zdravých kontrol po třech jídlech se stejným kalorickým obsahem ale různým složením – po běžném diabetickém jídle, po vegetariánském jídle a po jídle s nejvyšším obsahem tuků.

**Studijní skupina:** 60 diabetiků 2. typu z VEGA studie + 60 zdravých kontrol

**Zdraví dobrovolníci:**

    Kritéria pro zařazení:
    1. Ženy i muži ve věku 30-70 let

    2. Body Mass Index (kg/m^2^) nižší než 30

    3. Dobrovolník nesplňuje diagnostická kritéria metabolického syndromu – tzn. má maximálně 2 z těchto rizik. faktorů:

1. abdominální obezita – obvod pasu muži > 102 cm, ženy > 88 cm
2. krevní tlak léčený více než 2 antihypertenzivy nebo >130/85 mm Hg
3. cukrovka nebo porucha glukózové tolerance nebo glykémie nalačno > 5,6 mmol/l
4. HDL cholesterol – léčba nebo muži < 1 mmol/l, ženy < 1,3 mmol/l
5. Triglyceridy – léčba nebo > 1,7 mmol/l

4. Rodiče ani sourozenci nemají cukrovku

    Kritéria pro vyřazení:

    1. Současný alkoholismus nebo užívání drog

    2. Těhoteství, kojení

    3. Onemocnění štítné žlázy, onkologické onemocnění, nemoci srdce

4. Léčba inzulínem v minulosti

**Design studie a metody:** Randomizovaná studie. Účastníkům studie budou podávány jednotlivé snídaně v náhodném pořadí.

**Složení snídaní**:

1. **bageta Crocodille Sýrový mlsoun** – 180g, energie 452,8 Kcal/1895,7 kJ, složení: sacharidy 49,2 g (44,55%), proteiny 18,5 g (16,74%), lipidy 18,8 g (38,7%), z toho saturované 6,8 g, monoenové 6,0 g, polyenové 5,0 g.
2. **Kuskusový burger** 235 g, energie 455,88 Kcal /1907,4 kJ, složení: sacharidy 59,54 g (52,24%), proteiny 12,47 g (10,57% %), lipidy 18,85 g (37,31%), z toho saturované 6,2 g, monoenové 11,49 g, polyenové 28,26 g, vláknina 7,74 g, cholesterol  - 0,05 mg
3. **McCountry sendvič** - 455 kcal/1903,72 kJ, složení: sacharidy 31 g (27,25%), proteiny 24 g (21,1%), lipidy 26 g (51,43%), z toho saturované10 g (38,45%), vláknina 5 g

**Screening zdravých dobrovolníků**

1. Vyplnit **Screening (Příloha 4)**. Probrat všechna kritéria pro zařazení a pro vyřazení (bod po bodu)

2. Probrat s pacientem a podepsat **informovaný souhlas (Příloha 3)**

3. **Zvážit, změřit, spočítat BMI**

4. Změřit **TK**

5. **Odběry** - Na, K, Cl, urea, kreatinin, bilirubin, ALT, AST, GMT, HbA1c, HIV, HbsAg, chol, TG, HDL chol, LDLchol, glykémie, KO

**Plán pro jednotlivé návštěvy (1,2,3):**

1. Probrat s pacientem a podepsat **informovaný souhlas (Příloha 1)**

2. **Zvážit, změřit obvod pasu, obvod boků**

3. **Meal test**  test pro stanovení glukózové tolerance a sekrece inzulinu po standardní snídani

**Odběry.**

Glykémie – 0, 30, 60, 120 a 180 min.

IRI – 0, 30, 60, 120 a 180 min.

C-peptid– 0, 30, 60, 120 a 180 min.

TG, NEMK – 0, 30, 60, 120 a 180 min.

GI peptidy - **speciální zkumavka s inhibitorem proteinázy DPP-IV** – 0, 30, 60, 120 a 180 min.

Ox. Stes – **1 velká fialová + 1 malá červená s gelem** – 0,120,180 min. – ad Z6 (mgr. Malínská)

2x zásobní sérum - 0, 30, 60, 120 a 180 min.

Při první bagetě pacienti z VEGA studie:

cholesterol, HDL-cholesterol, LDL-cholesterol, Apo A1, Apo B – 0´

HbA1c – 0‘ – šedá zkumavka

Fe, transferin, TIBC – **1 malá červená zkumavka**

hsCRP – 0´

4. L**ékařské vyšetření**

5. Pacient vyplní **dotazníky** (Příloha 9-14)

**Ukládání séra**

Ukládání séra a 1 ml do kepů pro skladování při -80 st. C

Jednotlivá séra budou ukládána do zvláštních krabiček.

Značení vzorků **přímo na kep**: Pořadové číslo a příjmení (Exp.skupina 1-39E, Kontrolní skupina 51-83K, Zdraví 100-160 Z)

Příklad: 1E Mullerová

**na krabičku: Název vyšetření (př. IL-6)**

Pořadové číslo a příjmení uložených pacientů

|  | 1 | 2 | …10 |
| --- | --- | --- | --- |
| A | 1E M | 1EM |  |
| B |  |  |  |
| C |  |  |  |

…J

**Přílohy:**

1. Informovaný souhlas

2. Screening

3. Vyšetření 6m foolow-up

9. Dotazník kvality života OWLQOL

10. Baeckeho dotazník habituální pohybové aktivity

11. Mezinárodní dotazník na míru fyzické aktivity IPAQ short

12. Dotazník jídelních zvyklostí (Stunkard, Messick)

13. Dotazník na míru depresivity dle Becka

14. Graf váhy

15. Týdenní jídelníček

Personální zajištění:

1. Lékaři - Hana Kahleová, Simona Kratochvílová, Katka Zídková, Tomáš Neškudla

2. LKP - Dáša Šišáková, Dana Kobrová, Dana Lapešová, Jitka Purrová, Blažena Vodičková

3. Dietní sestry – Vlaďka Havlová, Růžena Milatová
